# Sports Nutrition: Current and Novel Insights

**DOI:** 10.3390/nu17091420

**Published:** 2025-04-23

**Authors:** David C. Nieman

**Affiliations:** Human Performance Laboratory, Department of Biology, North Carolina Research Campus, Appalachian State University, Kannapolis, NC 28081, USA; niemandc@appstate.edu; Tel.: +1-828-773-0056

This Special Issue invited the submission of sports nutrition-based manuscripts that advanced scientific understanding and provided novel insights on all aspects of performance and recovery. Exercise performance and recovery can be enhanced through prudent training- and nutrition-based approaches. The early history of sports nutrition was based on the importance of increased intake of carbohydrates and adequate hydration. Sports nutrition has entered a new era of investigation due to advances in technology, research designs, and data computational methods. In multi-omics studies, investigators utilize technologies from genomics, transcriptomics, proteomics, metabolomics, and epigenomics to gain a comprehensive understanding of biological systems. Human system biology approaches based on multi-omics outcomes have revealed significant influences of a wide variety of specific foods and supplements on physiological responses to exercise that were previously ignored [1,2].

As emphasized by Bedrač et al. in a scoping review [1], the number of multi-omics-based sports nutrition studies is expanding, but most are focused on discovering novel biomarkers. There is little evidence that these are linked significantly to athletic endeavors and long-term health. Multi-omics outcomes are a key component of precision sports nutrition that seeks to optimize nutrition guidelines at the individual athlete level for long-term performance and health. However, precision sports nutrition is currently considered an emerging science that will require continued advancement before it becomes practical and advantageous at the individual athlete level [2].

Two studies in this Special Issue used multi-omics technologies to investigate the effects of cranberry juice or mango ingestion on recovery from long endurance cycling [3,4]. Both studies were based on the concept that 2–4 weeks of ingestion of polyphenol-rich fruits or juices would significantly increase the generation of gut-derived metabolites that mitigate exercise-induced inflammation and innate immune system perturbations. The polyphenol profiles in cranberries and mangoes are distinctive, and intake of these fruits resulted in a strong gut-derived phenolic signature. In both studies, the 2.25 h cycling bouts increased post-exercise inflammation, but no countermeasure effect was measured after fruit ingestion. Fruits vary widely in the types and amounts of polyphenols, and more research is needed to determine the most effective fruits for countering inflammation in athletes following prolonged and intensive exercise [4,5].

The gut microbiome composition plays an important role in regulating human health and disease [6]. Probiotics, prebiotics, and postbiotics have the potential to modulate the gut microbiota in different ways. Probiotics are live microorganisms in cultured foods such as yogurt and kefir; prebiotics are nondigestible food components such as dietary fiber that act as substrates for gut bacteria; and postbiotics are the active substances produced by probiotics during their growth, such as short-chain fatty acids in miso [7]. In a systematic review, Kerksick et al. [8] summarized the published evidence linking postbiotic supplementation and exercise performance and recovery. This is a nascent area of investigation, and limited evidence from nine studies suggested some advantages in postbiotic supplementation for athletes, including positive influences on mood and fatigue.

Melatonin is a hormone produced by the brain’s pineal gland that plays a role in regulating the body’s sleep–wake cycle. While known for its role in sleep, melatonin also exerts antioxidant and anti-inflammatory influences and may play a role in other areas of immunity, health, and performance [9]. In a review of eight studies focused on professional football athletes, Almendros-Ruiz et al. [10] concluded that melatonin supplementation may attenuate exercise-induced oxidative stress, inflammation, and muscle damage, but that more research is needed on an effective dosing strategy.

Nitrate supplements, particularly beetroot juice, are popular among athletes for their performance benefits [11]. In a survey by Sebastiá-Rico et al. [12] of dietitians and nutritionists from 45 teams in Spanish football leagues, 56% reported providing nitrate supplements to the athletes before matches. Supplement dosing regimens, however, varied widely between the nutrition professionals, and few cautioned against the use of mouthwashes.

Creatine is made by the human body and converted into creatine phosphate and stored in the muscles, where it is used to fuel intensive muscle contractions. Although found in meat, creatine supplements are popular among bodybuilders and competitive athletes. Creatine supplementation has a small but significant effect on strength and power [13]. Limited data suggest that creatine may impact sleep in different ways and help reduce fatigue and tiredness. In a 6-week randomized clinical trial, Cruz et al. [14] showed that creatine versus placebo supplementation increased sleep duration but not sleep quality during resistance training in females. More research is needed to confirm this potential benefit of creatine supplementation for athletes during intensive training.

Para-cycling is a Paralympic sport that incorporates cycling events for athletes with disabilities, including those with visual and physical impairments. Few nutrition-based studies have been published with paracyclists, and the descriptive study by Shaw et al. [15] reported the nutrient intakes and diet quality of 31 paracyclists using food frequency questionnaires. As previously reported in other athletic groups, most micronutrients were consumed at or above recommended levels.

High-intensity interval exercise (HIIT) is a valuable, time-efficient training system for individuals with type 1 diabetes and has been linked in previous studies to improved cardiorespiratory fitness and insulin sensitivity [16]. The study by Hall et al. [17] confirmed that HIIT was effective in improving short-term glycemic control.

This Special Issue on current and novel insights in sports nutrition provided nine excellent articles that advanced the scientific understanding of the use of fruit and various types of supplements on performance and metabolic recovery, and the value of interval training for individuals with type 1 diabetes.
